# The Complete Mitogenome of *Pyrrhocoris tibialis* (Hemiptera: Pyrrhocoridae) and Phylogenetic Implications

**DOI:** 10.3390/genes10100820

**Published:** 2019-10-18

**Authors:** Qi-Lin Zhang, Run-Qiu Feng, Min Li, Zhong-Long Guo, Li-Jun Zhang, Fang-Zhen Luo, Ya Cao, Ming-Long Yuan

**Affiliations:** 1 State Key Laboratory of Grassland Agro-ecosystems; Key Laboratory of Grassland Livestock Industry Innovation, Ministry of Agriculture and Rural Affairs; Engineering Research Center of Grassland Industry, Ministry of Education; College of Pastoral Agriculture Science and Technology, Lanzhou University, Lanzhou 730000, China; zhangqilin88888@126.com (Q.-L.Z.);; 2 Faculty of Life Science and Technology, Kunming University of Science and Technology, Kunming 650500, China

**Keywords:** insects, true bugs, mitochondrial DNA, phylogeny

## Abstract

We determined the complete mitogenome of *Pyrrhocoris tibialis* (Hemiptera: Heteroptera: Pyrrhocoridae) to better understand the diversity and phylogeny within Pentatomomorpha, which is the second largest infra-order of Heteroptera. Gene content, gene arrangement, nucleotide composition, codon usage, ribosomal RNA (rRNA) structures, and sequences of the mitochondrial transcription termination factor were well conserved in Pyrrhocoroidea. Different protein-coding genes have been subject to different evolutionary rates correlated with the G + C content. The size of control regions (CRs) was highly variable among mitogenomes of three sequenced Pyrrhocoroidea species, with the *P. tibialis* CR being the largest. All the transfer RNA genes found in Pyrrhocoroidea had the typical clover leaf secondary structure, except for trnS1 (AGN), which lacked the dihydrouridine arm and possessed an unusual anticodon stem (9 bp vs. the normal 5 bp). A total of three different phylogenetic relationships among the five super-families of Pentatomomorpha were obtained using three analytical methods (MrBayes and RAxML under site-homogeneous models and PhyloBayes under a site-heterogeneous CAT + GTR model) and two mitogenomic datasets (nucleotides and amino acids). The tree topology test using seven methods statistically supported a phylogeny of (Aradoidea + (Pentatomoidea + (Lygaeoidea + (Pyrrhocoroidea + Coreoidea)))) as the best topology, as recognized by both RAxML and MrBayes based on the two datasets.

## 1. Introduction

Pentatomomorpha (Insecta: Hemiptera) is the second largest among the seven infra-orders of Heteroptera, with >14,000 species in 40 approximate families [1]. Most of Pentatomomorpha insects are phytophagous and major pests in agriculture, forestry, and livestock, while a few species of them are bloodsucking or predatory [1]. Pentatomomorphahas been divided into five super-families: Aradoidea, Pentatomoidea, Coreoidea, Lygaeoidea, and Pyrrhocoroidea. The super-families, except the Aradoidea, are grouped as Trichophora [1,2]. During the past several decades, phylogenetic relationships among the five super-families within Pentatomomorpha have been extensively explored based on the morphological and molecular data. For example, using morphological evidence, Henrysuggested a relationship (Aradoidea + (Pentatomoidea+ ((Coreoidea + Pyrrhocoroidea) + (Idiostoloidea+ Lygaeoidea)))) [3]. Using molecular evidence, Xie et al. performed Bayesian analysis with the 18S ribosomal DNA (rDNA) dataset on the main lineages of Trichophora, which givesa hypothesis of (Pentatomoidea + (Pyrrhocoroidea + (Coreoidea+ Lygaeoidea))) [4]. Based on the partial sequence of *cox1* and 18S rDNA, Li et al. proposed a hypothesis of (Aradoidea + (Pentatomoidea+ (Pyrrhocoroidea+ Coreoidea + Lygaeoidea))). However, Li et al. found that the super-families Pyrrhocoroidea, Coreoidea, and Lygaeoidea are not monophyletic [5]. To date, the hypothesis (Aradoidea + (Pentatomoidea + the remainder of Trichophora)) has been accepted by most researchers [4,5,6]. However, phylogenetic relationships among the three super-families within Eutrichophora (Coreoidea, Pyrrhocoroidea, and Lygaeoidea) are still controversial [3,4,5,7,8,9].

Mitochondrial genomes (mitogenomes) are the most extensively used genetic markers for evolutionary and population genetics studies of insects [10,11,12,13]. At present, many mitogenomes of Pentatomomorpha are available in GenBank, but most sequenced mitogenomes are from Pentatomoidea, while only two species were sequenced for Pyrrhocoroidea. Phylogenetic results based on mitogenomic data supported the monophyly of each of the five super-families, but phylogenetic relationships of Eutrichoideawere not well resolved. Most studies supported the sister-group relationship between the Pyrrhocoroidea and Coreoidea [6,7], but several studies supported a closer relationship between the Lygaeoidea and Coreoidea [5,14]. A small sampling size of Pyrrhocoroidea likely caused incongruent phylogenetic relationships within Eutrichophora, which may have influenced the accuracy of the result and created uncertainty to true evolutionary relationships [15]. Phylogenetic analyses using concatenation of genomic-scale data have been seen as the panacea for resolving the incongruences among inferences based on a few genes or single genes [16]. However, phylogenomics may also suffer from systematic errors, due to the cumulative effects of saturation [17], among-taxa compositional (GC content) heterogeneity [18,19], or the codon-usage bias (distinct preferences for alternative synonymous codons) [20].

In this case, we sequenced and annotated the complete mitogenome of *Pyrrhocoris tibialis* (Hemiptera: Pyrrhocoridae), which represents the third sequenced species from the Pyrrhocoroidea. Pyrrhocoroidea (Pentatomomorpha) consists of about 400 known species in 65 genera, and most members of Pyrrhocoroidea are phytophagous, and economically important in agriculture [21]. On the basis of annotation of the *P. tibialis* mitogenome, a comparative mitogenomic analysis was performed for the three species of Pentatomoidea. Combined with 24 Pentatomomorpha mitogenomes from NCBI (https://www.ncbi.nlm.nih.gov/), two mitogenomic datasets (nucleotide and amino acid) and three atypical methods (RAXML, MrBayes, and PhyloBayes) were used to reconstruct phylogenetic relationships among the five super-families within Pentatomomorpha.

## 2. Materials and Methods

### 2.1. Sampling, DNA Extraction, and Sequencing

Adult specimens of *P. tibialis* were collected from alfalfa in Yuzhong County, Lanzhou City, Gansu Province, China, on 5 June 2014. Samples and voucher specimens are deposited in the State Key Laboratory of Grassland Agro-Ecosystem, College of Pastoral Agricultural Science and Technology, Lanzhou University in Lanzhou, China. All specimens were initially preserved in 100% ethanol in the field, and transferred to −20 °C until used for DNA extraction. The total genomic DNA was extracted from thorax muscle of a single specimen using the insect genome DNA extraction kit (Omega, Norcross, GA, USA), according to the manufacturer’s protocols. Polymerase chain reactions (PCRs) were performed with the LA PCR Kit (TaKaRa, Shiga, Japan) following the manufacturer’s recommendations. The primers and their sources are presented in Table S1. PCR products were electrophoresed in 1.5% agarose gel, purified with an EasyPure PCR Purification Kit (TransGen Biotech, Beijing, China), and then both strands were sequenced with primer walking on an ABI 3730 automated DNA sequencer (Applied Biosystems, Carlsbad, CA, USA).

Failed and unsatisfactory sequencing fragments were cloned into the pEASY-T1 vector (TransGen Biotech, Beijing, China), and then conducted bidirectional sequencing.

### 2.2. Annotation and Sequence Analysis

To ensure the accurate reading of the sequence, the sequencing results were manually corrected, which ensures the consistency of base recognition and an original peak map. After removing the vector and primer sequences, the bidirectional sequencing results of each PCR fragment were assembled to obtain the correct sequence of the target fragment using BioEdit 7.2 (https://bioedit.software.informer.com/). Mitochondrial protein-coding genes (PCGs), ribosomal RNA genes (rRNAs), and transfer RNA (tRNA) genes of *P. tibialis* were annotated by using the methods described in our previous studies [7,22]. MEGA 6.06 [23] was used to analyze base composition, codon usage, synonymous mutations (*Ks*), and non-synonymous mutations (*Ka*). Asymmetry of base composition between two chains was calculated as the following formula [24]:

AT-deflection = [A − T]/(A + T); GC-deflection = [G − C]/[G + C],(1)

### 2.3. Phylogeneticsand Analyses of Sequence Heterogeneity

Twenty-eight Pentatomomorpha species with complete or nearly complete mitogenomes were used in phylogenetic analyses, which represented five super-families and 17 families. Two species of Cimicomorpha, *Lygus lineolaris* and *Apolygus lucorum*, were used as outgroups. Details of the species used in this study are listed in Table S2. The complete sequences of each gene were used for phylogenetic analysis (excluding stop codons of the PCGs). All PCGs were aligned based on amino acid sequence alignments in MAFFT (http://mafft.cbrc.jp/alignment/server/). The aligned sequences were concatenated as two matrices used in phylogenetic analyses: (1) The P123 matrix, including all the three codon positions of PCGs, and (2) the amino acid (AA) matrix, including the amino acid sequence of PCGs. Next, GBlocks [25] was used to remove vacancies and blur sites for two aligned sequence matrices. The aligned single PCGs were combined to obtain the final mitochondrial gene set. DAMBE analysis [26] results showed that the three codon sites of 13 PCGs were not significantly saturated (Table S3). All codons, thus, were used in phylogenetic analysis. PartitionFinder 1.1.1 [27] was adopted to test the optimal partition and evolutionary model for sequence data, and the results were used for downstream phylogenetic analyses (Table S4).

Subsequently, for the nucleotide (P123) and amino acid (AA) datasets of 13 PCGs, three methods (RAxML, MrBayes, and PhyloBayes) were used to construct phylogenetic relationships within Pentatomomorpha. All phylogenetic analyses were performed on the CIPRES Science Gateway 3.3 [28] online platform. Machine learning analysis was performed using the GTRGAMMAI model in RAxML-HPC2 on XSEDE 8.0.24 [29], and the branch reliability was evaluated with 1000 rapid bootstraps. Bayesian analysis was implemented using MrBayes 3.2.2 [30], with four independent Markov chains, including three hot chains and one cold chain, running a 1 × 10^8^ generation simultaneously. For every 1000 runs of collection sampling, when estimated sample size is greater than 100 and the potential scale reduction factor is close to 1.0. The two analytical processes are considered to be a stable state. Removing 25% aged samples, the remaining samples were used to construct the 50% consensus tree, and the Bayesian posterior probability (BPP) was calculated. Another Bayesian analysis under a site-heterogeneous model was implemented using PhyloBayes MPI 1.5a on the CIPRES webserver [28]. After removing constant sites from the alignment, two independent chains starting from a random tree were run under the CAT + GTR model.

The heterogeneity of sequence divergence within the two datasets (AA and P123) was analyzed using AliGROOVE [31] with the default sliding window size. Indels in the nucleotide datasets were treated as ambiguities and a BLOSUM62 matrix was used as the default amino acid substitution matrix. The metric establishes pairwise sequence distances between the individual terminal branch or subclades with the terminal branch outside of the focal group. The distances were then compared to distances over the entire data matrix. The metric values can vary between -1, if distances are very different from the average for the entire data matrix to +1 for distances that match the average for the entire matrix. This provides an indirect measure of heterogeneity of a given sequence or clade with respect to the full dataset.

Because of the inconsistency of phylogenetic relationships obtained by different data sets and phylogenetic analytical methods, in order to determine which phylogenetic tree is trustworthy, the IQ-TREE web server (http://iqtree.cibiv.univie.ac.at/) was used to test the tree topological structure [32]. The data sets of P123 and AA were analyzed by KH (Kishino-Hasegawa test) [33], SH (Shimodaira-Hasegawa test) [34], WKH (weighted Kishino-Hasegawa test) [33], WSH (weighted SH test) [34], ELW (Expected Likelihood Weight) [35], and AU (approximately unbiased test) [36]. Furthermore, 1000 replicates were set.

## 3. Results and Discussion

### 3.1. Genome Organization

**Figure 1:**
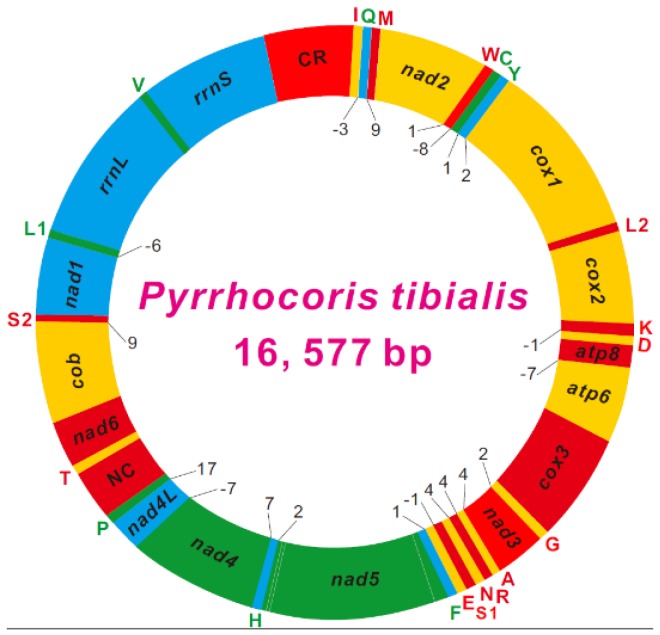
Circular map of the mitochondrial genome of *Pyrrhocoris tibialis*. Protein coding and ribosomal genes are shown with standard abbreviations. Genes for transfer RNAs (tRNAs) are abbreviated by a single letter, with S1 = AGN, S2 = UCN, L1 = CUN, and L2 = UUR. Genes coded in the *J* (H)-strand (clockwise orientation) are red-colored or orange-colored. Genes coded in the *N* (L)-strand (counter-clockwise orientation) are green-colored or cyan-colored. Numbers at gene junctions indicate the length of small non-coding regions where negative numbers indicate an overlap between genes.

The *P. tibialis* mitogenome was a typical closed-circular DNA molecule with 16,577 bp in size, and contained 37 typical mitochondrial genes, i.e., 13 PCGs, 22 tRNAs, and two rRNAs (Figure 1 and Table S5). The size of the *P. tibialis* mitogenome was similar that of the same-family species *Dysdercus cingulatus* (16,249 bp), but much larger than *Physopelta gutta* (Largidae, 14,935 bp). The size difference among the three pyrrhocorid mitogenomes was primarily due to variable length of the putative control region (CR), which ranges from 224 bp in *P. gutta* to 1620 bp in *P. tibialis*. The *P. tibialis* mitogenome is highly compact in genome size as that in other animals, with seven gene overlapping regions involved in a total of 33 nucleotides. The largest gene overlapping region (8 bp) is located between *trnW* and *trnC*, as observed in the other two Pyrrhocoroidea species [2]. Except for a large non-coding region (mitochondrial CR), there were 14 small non-coding intergenic regions. The intergenic spacer between *trnP* and *trnT* was 345 bp. The remaining 13 regions ranged from 1 bp to 9 bp, with a total of 63 bp.

**Figure 2:**
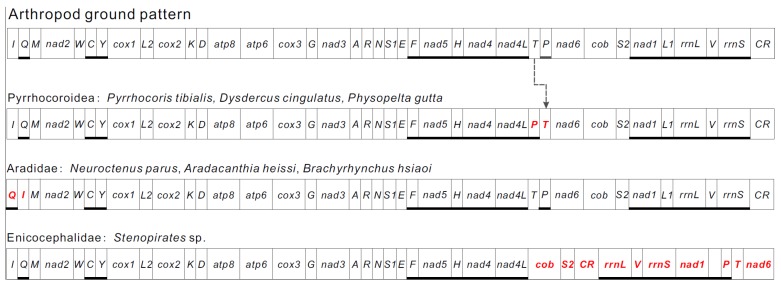
Mitochondrial gene rearrangement among Heteroptera. Abbreviations of gene names follow Figure 1. Genes are transcribed from left to right except those underlined, which have the opposite transcriptional orientation. Rearrangements of mitochondrial genes were highlighted by a red color.

With some notable exceptions, within the class Insecta, the order of the mitochondrial genes is highly conserved and has led to the proposal of an ancestral gene order. A translocation that *trnT* was followed by *trnP* was observed in the mitogenome of three species of Pyrrhocoroidea. Comparing with the original gene order in mitogenomes of arthropods, the original *trnT*-*trnP* is rearranged as *trnP*-*trnT* in the *P. tibialis* mitogenome, and there was a large non-coding sequence between *trnP* and *trnT* (Figure 1 and Figure 2). This rearrangement was also observed in the other two pyrrhocorid species, which indicates that this kind of T–P rearrangement is specific to the Pyrrhocoroidea. Due to the unique sort of model of mitochondrial genes in the Pyrrhocoroidea, we speculate that this phenomenon in Pyrrhocoroidea likely occurred after the Pyrrhocoroidea differentiated from ancestors. The gene rearrangement was seldom discovered in Heteropteraso far. It was limited to Aradoidea, Reduvioidea, and Pyrrhocoroidea. On the contrary, gene rearrangements are common in Auchenorrhyncha.

The unexpected non-coding region between *trnP* and *trnT* was further verified by PCR amplification and sequencing using species-specific primers. Non-coding regions were also found in the other two pyrrhocorid species, despite their relatively small size ranging from 1 bp to 60 bp in *P. gutta* and *D. cingulatus*, respectively. This rearrangement can be explained by the duplication-random deletion model that is frequently used to explain the diversity of gene rearrangements in metazoan mitogenomes [37,38]. This model considers that some genes first use slipped-strand mispairing to produce a repetitive gene, and then delete a redundant gene or transfer to a pseudogene randomly during the process of duplication. Thus, the model can explain gene translocation, the small non-coding region generated around the start point, length heterogeneity, the extreme variation of the replacing rate, and a repetitive copy of the tRNA gene.

### 3.2. Nucleotide Composition and Codon Usage

The nucleotide composition of the *P. tibialis* mitogenome was significantly biased toward A and T. The total A + T content of the *J* (H)-strand was 75.9%, which is similar to that of the other two pyrrhocorid species (Table 1). Among 13 PCGs, *cox1* had the lowest A + T content (69.02%), while the highest was 86.27% in *atp8*. The analysis of the nucleotide composition at each codon position of the concatenated 13 PCGs of *C. tetraspilus* demonstrated that the third codon position (86.19%) had an A + T content higher than that of the first (70.34%) and second (67.46%) positions. The similar nucleotide composition patterns were also observed in other Coreoidea species [7]. Similar to other sequenced Pentatomomorpha insects, the base composition of the *P. tibialis* mitogenome was clearly biased toward A + T (Table 1). The A + T content of the *J* (H)-strand in the *P. tibialis* mitogenome was 75.91%, with 75.15% in 13PCGs, 79.94% in *rrnL*, 77.91% in *rrnS*, and 73.46% in the CR. The A + T content of three codon sites in 13 PCGs was significantly different, with the third codon site showing much higher A + T content than that of the first and the second sites. AT-skew of the *J* (H)-strand in the *P. tibialis* mitogenome was positive (0.101), while the GC-skew was negative (−0.181).

**Figure 3:**
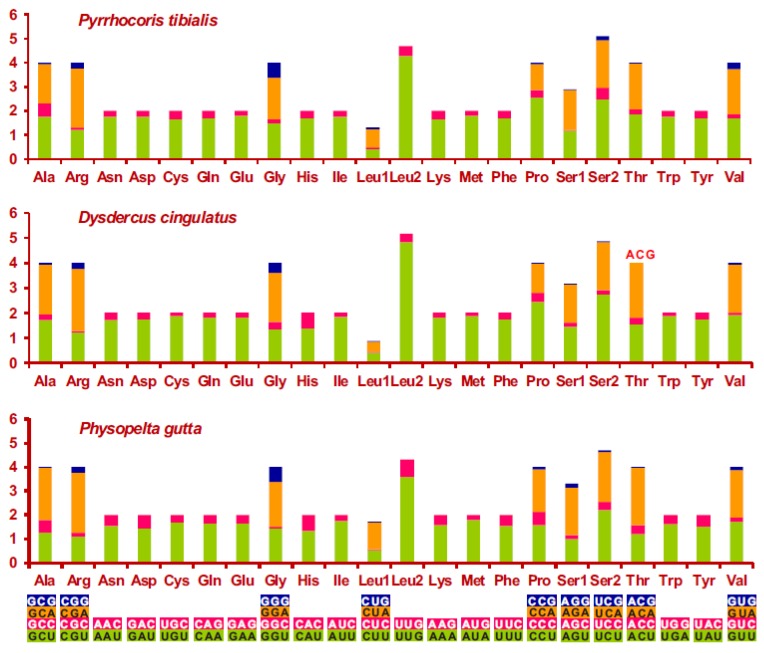
Relative synonymous codon usage (RSCU) in the mitochondrial genomes of three *Pyrrhocoroidea* species. Codons that are not present in the genome are indicated in red. Codon families are provided on the *X*-axis. RSCU: Ratio of the actual number of synonymous codons used to translate specific amino acids to the expected number. When the observed values of synonymous codons are the same as the expected values, RSCU = 1, and the codons are not biased. When RSCU > 1, the codons are positively biased. When RSCU < 1, the codons are negatively biased.

The high A + T content and nucleotides bias in the *P. tibialis* mitogenome were also reflected in codon usage of PCGs (Figure 3). Relative synonymous codon usage (RSCU) analysis indicated that all the 62 mitochondrial codons of invertebrates were used in the *P. tibialis* mitogenome. However, the appearance frequency of codons that ended with A or T was much higher than that of the other synonymous codons, which indicates that codons with rich AT content were frequently used (Figure 3). Four AT-rich codons (TTT, TTA, ATT, and ATA) were the most frequently used codons in the *P. tibialis* mitogenome, which accounts for 31.8%. This pattern of codon usage in the *P. tibialis* mitogenome was highly similar to that of the other two species (*D. cingulatus* and *P. gutta*) within the super-family Pyrrhocoroidea.

### 3.3. Protein-Coding Genes

Except for *cox1* that started with ‘TTG’, the remaining 12 PCGs began with ‘ATN’ in the *P. tibialis* mitogenome. Seven of these used ‘ATA’ (*atp8*, *nad1*, *nad2*, *nad3*, *nad4L*, *nad5*, and *nad6*), and the other five genes started with ‘ATG’ (*atp6*, *cob*, *cox2*, *cox3*, and *nad4*) (Table S6). The six PCGs (*atp8*, *cob*, *nad2*, *nad3*, *nad4*, and *nad4L*) ended with ‘TAA’ in the *P. tibialis* mitogenome, and the remaining seven PCGs ended with incomplete termination codons. Two genes (*cox1* and *cox2*) ended with single ‘T’, and five genes (*atp6*, *cox3*, *nad1*, *nad5*, and *nad6*) ended with ‘TA’. Incomplete termination codons are common in insect mitogenomes. It is speculated that these incomplete termination codons can be completed by adding ‘A’ during transcription [39,40], and do not affect translation.

**Figure 4:**
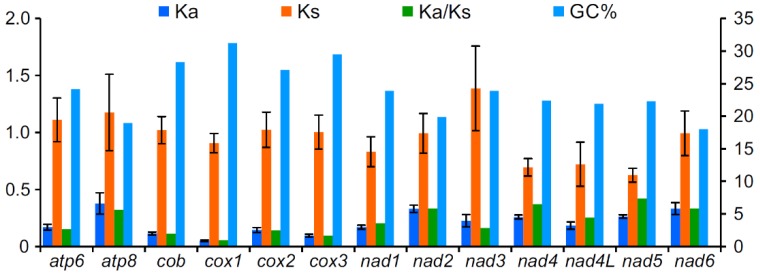
Evolutionary rates of 13 protein-coding genes in the mitochondrial genomes of three *Pyrrhocoroidea* species.

To analyze the evolutionary patterns of 13 PCGs in three species of Pyrrhocoridae, *Ka*, *Ks*, *Ka/Ks* and GC percentage of each PCG were calculated, respectively (Figure 4). The average *Ks* values of the three species were similar among 13 PCGs, and all values were around one. Conversely, *Ka* showed a great variation, of which *atp8* was the largest, which indicates that this gene had the fastest evolutionary rate. Moreover, *nad2* and *nad6* also presented a faster evolutionary rate, while *cox1*, *cox3*, and *cob* showed the slowest evolutionary rate. *Ka/Ks* values of all PCGs were less than 1 (<0.53), which suggested that these genes evolved under a strong purifying selection due to a functional constraint. *Ka/Ks* values of 13 PCGs were significantly negatively correlated with the GC percentage (*R*^2^ = 0.72, *p* < 0.01), which indicates that the variation of GC content may have caused different evolution patterns of mitochondrial PCGs. Alternatively, various evolutionary patterns promoted changes of GC content.

### 3.4. Ribosomal and Transfer RNAs

The size of *rrnL* and *rrnS* genes in the *P. tibialis* mitogenome were 1266 bp and 824 bp, respectively, and the content of AT was 79.94% and 77.91%, which were very similar to that of the other two pyrrhocorid species (Table 1). The total length of the 22 tRNA genes in the *P. tibialis* mitogenome was 1449 bp, among which the longest was 72 bp and the shortest is only 63 bp, with an average length of 66 bp. All 22 tRNAs can form a classical clover structure, including the TψC arm, the amino acid acceptor arm, the anticodon arm, and the dihydrouridine arm (Figure S1). Some of tRNA’s dihydrouridine (DHU) arm (*trnQ*, *trnY*, *trnC*, *trnG*, *trnF*, *trnH*, and *trnP*), amino acid acceptor arm (*trnQ*, *trnC*, *trnG*, *trnA*, and *trnF*), and anticodon arm (*trnH* and *trnT*) showed individual base mismatches, which was common in insect mitogenomes. Nevertheless, it was worth noting that the DHU arm of *trnS1* not only existed mismatches of G-U and A-C, but also the length of the stem was only 3 bp. The loop was only composed of three nucleotides, which suggested that the arm may not exist. In fact, the lack of a DHU arm in *trnS1* is common in sequenced mitogenomes of Metazoans [11]. DHU arms of *trnS1* seemed to be missed in many sequenced insects including the other two pyrrhocorid species [2].

### 3.5. Non-Coding Regions

The CR of the *P. tibialis* mitogenome was located between *rrnS* and *trnI*, with a length of 1620 bp and an AT content of 73.46% (Table S5). *P. tibialis* and *D. cingulatus* had similar CR length (1620 bp and 1617 bp), but CR of *P. gutta* was only 224 bp (Figure S2). The CRs of these three mitogenomes had tandem repeats, and there were two types of tandem repeats in *P. tibialis* and *D. cingulatus*, while there was only one type of tandem repeat sequence in *P. gutta*. There was a large non-coding region close to *rrnS* in *P. tibialis* and *D. cingulatus*, but it was not found in *P. gutta*. The absence of both non-coding regions close to *rrnS* and a small number of tandem repeats may be the reason that length of CR in *P. gutta* was significantly shorter than that of the other two species of the same super-family Pyrrhocoroidea.

### 3.6. Phylogenetic Analysis

**Figure 5:**
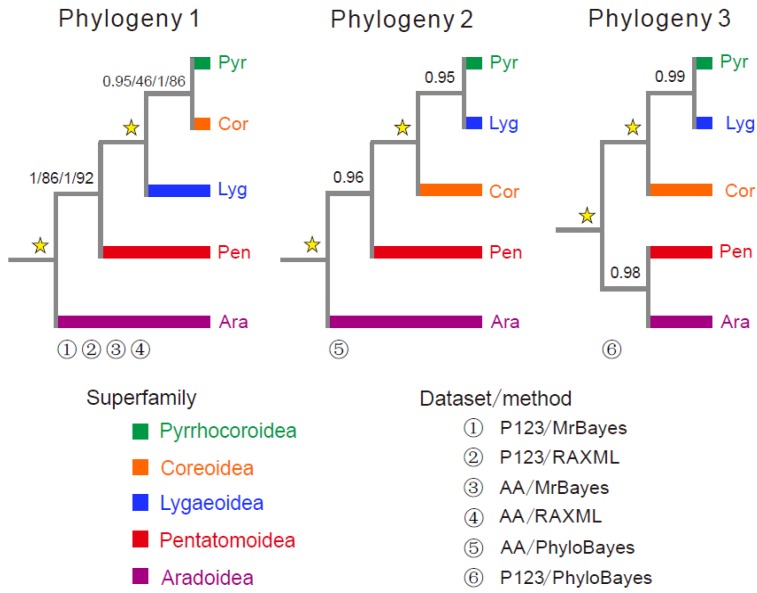
Phylogenetic relationships constructed by three phylogenetic methods (RAXML, MrBayes, and PhyloBayes) among five *Pentatomomorpha* super-families. Phylogenetic analyses are based on the concatenated nucleotide (P123) and amino acid (AA) sequences of 13 protein-coding genes. Numbers on branches are bootstrap values and Bayesian posterior probabilities. Numbers 1–6 indicate phylogenetic relationships among the five super-families obtained with different mitogenomic datasets and analytical methods.

Based on two datasets (P123 and AA) and three analytical methods (RAxML, MrBayes, PhyloBayes), a total of six phylogenetic trees were obtained (Figure 5, Figures S4–S9). These phylogenetic trees highly supported the monophyly of each of Pentatomoidea, Aradoidea, Lygaeoidea, Pyrrhocoridae, and Coreoidea, which has been revealed by previous studies [7,41]. A phylogeny of (Aradoidea+ (Pentatomoidea+ (Lygaeoidea+ (Pyrrhocoroidea + Coreoidea)))) was consistently supported by four phylogenetic analyses based on two methods (RAxML and MrBayes) and two datasets (Phylogeny 1 in Figure 5). However, phylogenetic trees constructed by the PhyloBayes method were different from that of both RAxML and MrBayes. The AA-tree was (Aradoidea + (Pentatomoidea + (Coreoidea + (Pyrrhocoroidea + Lygaeoidea)))), and the P123-tree was ((Aradoidea + Pentatomoidea) + (Coreoidea + (Pyrrhocoroidea + Lygaeoidea))). Our results indicated that the degrees of heterogeneity of the amino acid dataset were lower than those of the nucleotide datasets (Figure S4), as similarly reported in previous studies [19]. However, both AA and P123 datasets generally showed a high site-homogeneous pattern (or no significant site heterogeneity) within datasets used in this study (all AliGROOVE scores between two species > 0.0), despite a lower heterogeneity of the AA dataset than that of the P123 dataset. Furthermore, all of topology test results presented the greatest test values in phylogeny 1 based on both P123 and AA datasets (Table 2), which supports a phylogeny of (Aradoidea+ (Pentatomoidea+ (Lygaeoidea+ (Pyrrhocoroidea + Coreoidea)))). In general, the use of the CAT + GTR model, the site-heterogeneous mixture model (CAT-based model), implemented in PhyloBayes tends to reduce tree reconstruction artifacts [42,43,44], and then shows significant improvement over site-homogenous models in the reconstruction of the phylogeny of Heteroptera [14,19]. Nevertheless, in this study, due to a low heterogeneous sequence divergence between heteropteran mitochondrial AA and P123 datasets, low statistical values of topology tests were consistently presented in seven test methods using PhyloBayes. This observation suggested that robustness of tree topology was not improved by the CAT + GTR model of PhyloBayes. RAxML and MrBayes trees, which are phylogenetic analyses under a site-homogenous model, were presented to be stable. These trees consistently presented a phylogenetic relationship at the super-family level within Pentatomomorpha, which is similar to previous studies [7,14,19,45]. The trees were largely congruent with previous results based on the morphological data [6]. Therefore, compared to PhyloBayes, RAxML and MrBayes may be more plausible and phylogenetic within the suborder Pentatomomorpha, i.e., the phylogeny of (Aradoidea + (Pentatomoidea + (Lygaeoidea + (Pyrrhocoroidea + Coreoidea)))) was more reliable than the remaining two phylogenies obtained in this study.

Internal phylogenetic relationships within each of three super-families (Pentatomoidea, Aradoidea, and Pyrrhocoroidea) were stable, but internal phylogenetic relationships within Lygaeoidea and Pentatomoideawereunstable (Table S7). Within the super-family Lygaeoidea, phylogenetic relationships among five families (Malcidae, Lygaeidae, Geocoridae, Berytidae, and Colobathristidae) were variable depending on different analytical methods and datasets, which indicates the necessity of further study for phylogenetic relationships within Lygaeoidea. Three of six phylogenetic analyses (both P123 and AA trees constructed by MrBayes, P123 tree constructed by RAxML) consistently supported a phylogeny of ((Malcidae + Lygaeidae) + the other families) Figures S3–S8), while two AA-trees based on RAxML and PhyloBayes consistently presented a closer relationship between Lygaeidae and Geocoridae. Despite recent studies explored phylogenetic relationships within Lygaeoidea, only one family, Lygaeidae [19] or Geocoridae [14], was included in their analyses. For the two families Berytidae and Colobathristidae, which family was closer to the internal-branch families was still controversial, as reported in previous studies [14,19]. Herein, excluding the analysis based on P123 and PhyloBayes, the remaining five analyses consistently supported a closer phylogenetic relationship of the family Berytidae with internal branches within Lygaeoidea. Notably, it was difficult to demonstrate the phylogenetic location of Geocoridae in the super-family Lygaeoidea in this study, due to a severely confused topology between Geocoridae and the other families. Within the super-family Lygaeoidea, the phylogenetic location of Geocoridae was unstable. In the current study, only one species was included for each family (Geocoridae or Berytidae). However, Liu et al. added one species (*Metatropis longirostris*) belonging to the family Berytidae of phylogenetic trees in the super-family Lygaeoidea, which found that Geocoridae did not cluster together with Berytidae, but it was a sister lineage with the family Malcidae [19]. Therefore, the present study based on the limited taxa was difficult to well systematically infer the phylogenetic relationships at the family level, but several interesting issues involved in phylogenetics mentioned above within the super-family Lygaeoidea deserve to be further clarified in the future.

For the super-family Pentatomoidea, incongruent phylogenetic relationships were observed between topologies constructed based on the P123 and AA datasets. The former consistently presented phylogenetic relationships among two analytical methods (RAxML and MrBayes): ((Plataspidae, + (((Dinidoridae + Tessaratomidae) + Cydnidae) + Pentatomidae)) + Urostylididae), while the latter consistently exhibiteda phylogeny of (((Plataspidae + ((Dinidoridae + Tessaratomidae), Cydnidae)) + Pentatomidae) + Urostylididae). On account of lower degrees of heterogeneity of the AA dataset than that of the P123 dataset, it is, thus, speculated that phylogenetic relationships among families based on the nucleic dataset may be unfavorable in the current study. In addition, the PhyloBayes analysis based on the AA dataset consistently presented the same phylogenetic relationship with the AA-tree based on RAxML and MrBayes, which further supports (((Plataspidae + ((Dinidoridae + Tessaratomidae), Cydnidae)) + Pentatomidae) + Urostylididae) within Pentatomoidea in this study. Notably, the confusion of taxonomic relationships at the family level within *Pentatomoidea* needs to be further resolved with a denser taxa sampling by including molecular and morphological data in the future.

## 4. Conclusions

In conclusion, the complete mitogenome of *P. tibialis* was determined in this study, which further enriched the number of mitogenomes of *Pyrrhocoroidea*. We analyzed the main features of the *P. tibialis* mitogenome, and provided a comparative analysis with two other pyrrhocorid species. Furthermore, phylogenetic analyses were conducted for the five super-families within *Pentatomomorpha* based on nucleic and amino acid datasets. In this study, a low heterogeneity in base composition and contrasting evolutionary rates were detected among the five super-families within Pentatomomorpha, which results in no improvement of phylogenetic inference under a site-heterogeneous mixture model. Phylogenetic analyses based on mitogenomic data supported the monophyly of each super-family within Pentatomomorpha. Aphylogenetic relationship within this suborder was preliminarily proposed. This study is valuable for further understanding phylogenetic relationships among super-families within *Pentatomomorpha*. Notably, due to single species in several families and a single sequenced mitogenome per species, it likely limited the achievement of more phylogenetic information. Thus, to obtain more clarity, it is important to sequence more species per family and multiple mitogenomes per species in the future.

## Figures and Tables

**Table 1:** Nucleotide composition of *Pyrrhocoris tibialis* (PT), *Dysdercuscingulatus* (DC), and *Physopelta gutta* (PG).

	A + T%			AT-Skew			GC-Skew		
	PT	DC	PG	PT	DC	PG	PT	DC	PG
Whole genome	75.91	77.69	74.51	0.101	0.135	0.206	−0.181	−0.220	−0.207
Protein-coding genes	75.15	76.86	73.55	−0.134	−0.127	−0.099	0.020	0.015	−0.004
1st codon positions	69.46	71.62	69.01	−0.002	−0.010	0.033	0.220	0.240	0.198
2nd codon positions	67.88	68.09	67.61	−0.401	−0.408	−0.419	−0.106	−0.088	−0.100
3rd codon positions	88.10	90.86	84.02	−0.033	−0.009	0.051	−0.153	−0.325	−0.203
tRNA genes	76.69	78.44	77.46	0.020	0.035	0.028	0.142	0.162	0.138
*rrnL* genes	79.94	81.23	78.68	−0.103	−0.109	−0.204	0.260	0.286	0.257
*rrnS* genes	77.91	79.42	75.35	−0.115	−0.122	−0.175	0.297	0.256	0.323
Control region	73.46	79.22	75.45	−0.061	0.176	0.101	−0.219	−0.411	−0.309

**Table 2:** Topology test results.

Dataset	Topology	logL	KH	SH	WKH	WSH	ELW	AU
PCG	Phylogeny 1: ((((Pyr, Cor), Lyg), Pen), Ara)	−176,055.8	0.671	1	0.671	0.808	0.557	0.657
	Phylogeny 2: ((((Pyr, Lyg), Cor), Pen), Ara)	−176,060.0	0.329	0.581	0.329	0.554	0.205	0.386
	Phylogeny 3: (((Pyr, Lyg), Cor), (Pen, Ara))	−176,063.8	0.296	0.363	0.296	0.384	0.236	0.362
Amino acid	Phylogeny 1: ((((Pyr, Cor), Lyg), Pen), Ara)	−89,145.0	0.853	1	0.853	0.929	0.845	0.915
	Phylogeny 2: ((((Pyr, Lyg), Cor), Pen), Ara)	−89,157.7	0.147	0.295	0.147	0.254	0.133	0.135
	Phylogeny 3: (((Pyr, Lyg), Cor), (Pen, Ara))	−89,177.4	0.032	0.039	0.032	0.058	0.022	0.040

Pyr, Pyrrhocoroidea. Cor, Coreoidea. Lyg, Lygaeoidea. Pen, Pentatomoidea. Ara, Aradoidea. logL, log-likelihood. KH, Kishino-Hasegawa test [34]. SH, Shimodaira-Hasegawa test [35]. WKH, weighted KH test [34]. WSH, weighted SH test [35]. ELW, Expected Likelihood Weight [36]. AU, approximately unbiased test [37].

## Supplementary Materials

The following are available online at https://www.mdpi.com/2073-4425/10/10/820/s1. Table S1: PCR primers used in this study. Table S2: List of the species included in the present study. Table S3: Saturation test implemented in DAMBE. Table S4: The best partitioning schemes and substitution models selected by the PartitionFinder for the P123 and AA datasets. Table S5: Annotation and organization of the complete mitochondrial genome of Pyrrhocoris tibialis. Table S6: Start and stop codons and lengths of protein-coding genes (PCGs), and two rRNA genes from three Pyrrhocoroidea species. Table S7: Phylogenetic relationships within Lygaeoidea and Pentatomoidea based on different datasets and analytical methods. Figure S1: Putative secondary structures of the 22 tRNA genes identified in the mitochondrial genome of Pyrrhocoris tibialis. Figure S2: Organization of the A + T-rich region in Pyrrhocoroidea mitochondrial genomes. The location and copy number of tandem repeats are shown by the colored oval with Arabic numerals inside. Non-repeat regions are indicated by the colored box with the sequence size inside. Figure S3: Heterogeneous amino acid (A) and nucleotide (B) sequence divergence within heteropteran mitochondrial genomes. The mean similarity score between sequences is represented by a colored square, which ranges from −1 to +1. A lower value indicates a greater difference in rates from the remainder of the data set, i.e., heterogeneity (red coloring), while a greater value indicates rates that match all other comparisons (blue coloring). Figure S4: MrBayes phylogenetic relationships among five Pentatomomorpha super-families. Phylogenetic analysis is based on the concatenated nucleotide sequences of 13 mitochondrial PCGs. Numbers on branches are Bayesian posterior probabilities. Figure S5: RAxML phylogenetic relationships among five Pentatomomorpha super-families. Phylogenetic analysis is based on the concatenated nucleotide sequences of 13 mitochondrial PCGs. Numbers on branches are bootstrap values. Figure S6: PhyloBayes phylogenetic relationships among five Pentatomomorpha super-families. Phylogenetic analysis is based on the concatenated nucleotide sequences of 13 mitochondrial PCGs. Numbers on branches are Bayesian posterior probabilities. Figure S7: MrBayes phylogenetic relationships among five Pentatomomorpha super-families. Phylogenetic analysis is based on the concatenated amino acid sequences of 13 mitochondrial PCGs. Figure S8: RAxML phylogenetic relationships among five Pentatomomorpha super-families. Phylogenetic analysis is based on the concatenated amino acid sequences of 13 mitochondrial PCGs. Figure S9: PhyloBayes phylogenetic relationships among five Pentatomomorpha super-families. Phylogenetic analysis is based on the concatenated amino acid sequences of 13 mitochondrial PCGs.
