# Supplementary material for: The Complete Mitogenome of Pyrrhocoris tibialis (Hemiptera: Pyrrhocoridae) and Phylogenetic Implications
Source: Genes (Basel). 2019 Oct 18;10(10):820. doi: 10.3390/genes10100820 (PMC6826757; doi:10.3390/genes10100820)
Supplement: Supplementary file 1 [file genes-10-00820-s001.zip › Figure S1.pdf]

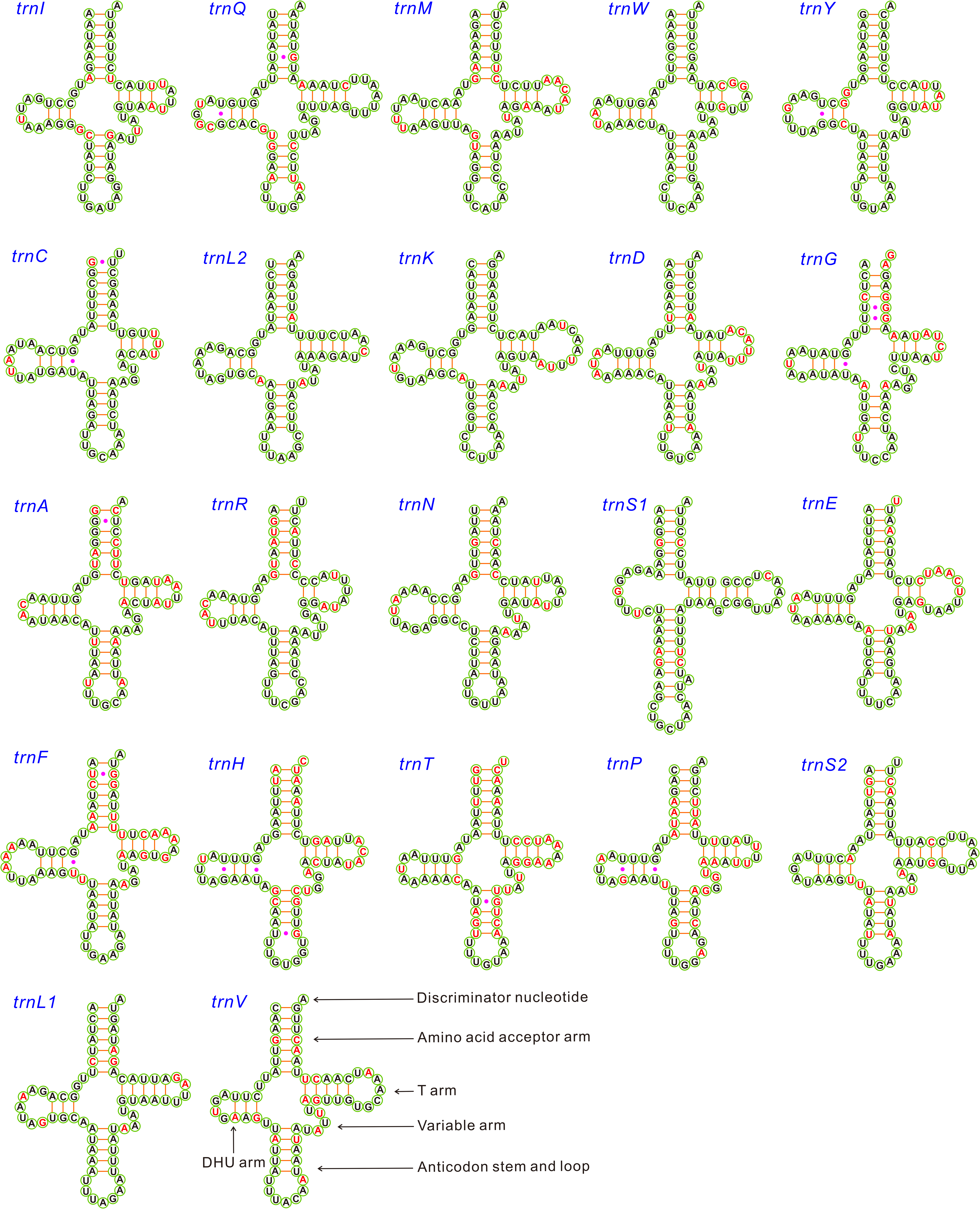
trnI
trnQ
trnM
trnW
trnY
trnC
trnL2
trnK
trnD
trnG
trnA
trnR
trnN
trnS1
trnE
trnF
trnH
trnT
trnP
trnS2
trnL1
trnV
Discriminator nucleotide
Amino acid acceptor arm
T arm
Variable arm
DHU arm
Anticodon stem and loop
