# Supplementary material for: The Complete Mitogenome of Pyrrhocoris tibialis (Hemiptera: Pyrrhocoridae) and Phylogenetic Implications
Source: Genes (Basel). 2019 Oct 18;10(10):820. doi: 10.3390/genes10100820 (PMC6826757; doi:10.3390/genes10100820)
Supplement: Supplementary file 1 [file genes-10-00820-s001.zip › Table S1-S7.docx]

Table S1 PCR primers used in this study.

| Fragment | Primer name | Sequence (5’-3’) | Reference |
| --- | --- | --- | --- |
| 1 | C1-J1709 | AATTGGWGGWTTYGGAAAYTG | Simon et al., 2006 |
|  | C1-N2776 | GATAATCTGAGTATCGWCGNGG | Simon et al., 2006 |
| 2 | C1-J2756 | ACATTCTTTCCTCARCAYTT | Simon et al., 2006 |
|  | C2-N3665 | CCACAAATTTCTGAACATTG | Simon et al., 2006 |
| 3 | DPSA-F1 | CAGGACGGCTAAATCAAGGA | this study |
|  | DPSN-R1 | CTCGTGGCTTTAGCAGCTTT | this study |
| 4 | N5-J7572 | AAAGGGAATTTGAGCTCTTTTWGT | Simon et al., 2006 |
|  | N4-N8727 | AAATCTTTRATTGCTTATTCWTC | Simon et al., 2006 |
| 5 | N4-J8641 | CCAGAAGAACATAANCCRTG | Simon et al., 2006 |
|  | N4L-N9629 | GTTTGTGAGGGWGYTTTRGG | Simon et al., 2006 |
| 6 | DPSF-F1 | AATCATTACCATAACCACGAA | this study |
|  | DPSI-R1 | ATGTTGCACAGGCTTAGGTG | this study |
| 7 | N1-J12261 | TACTTCATAAGAAATAGTYTGRGC | Simon et al., 2006 |
|  | LR-N13000 | TTACCTTAGGGATAACAGCGTAA | Simon et al., 2006 |
| 8 | LR-J12888 | CCGGTTTGAACTCARATCATGTA | Simon et al., 2006 |
|  | SR-N14220 | ATATGYACAYATTGCCCGTC | Simon et al., 2006 |
| 9 | DPSJ-F1 | AATCCAACTCCACCTTCCAAT | this study |
|  | DPSD-R1 | CCAGTTCCTGCCCCTATTTC | this study |

Table S2 List of the species included in the present study.

| Infraorder | Superfamily/family | Species | Size (bp) | Accession number | Reference |
| --- | --- | --- | --- | --- | --- |
| Cimicomorpha | Miroidea |  |  |  |  |
|  | Miridae | *Lygus lineolaris* | 14,239 | KU234540* | Direct Submission |
|  |  | *Apolygus lucorum* | 14,768 | NC_023083 | (Wang et al., 2014) |
| Pentatomomorpha | Aradoidea |  |  |  |  |
|  | Aradidae | *Aradacanthia heissi* | 15,528 | HQ441233 | (Shi et al., 2012) |
|  |  | *Brachyrhynchus hsiaoi* | 15,250 | NC_022670 | (Li et al., 2015a) |
|  |  | *Neuroctenus parus* | 15,354 | NC_012459 | (Hua et al., 2008) |
|  | Coreoidea |  |  |  |  |
|  | Alydidae | *Riptortus pedestris* | 17,191 | NC_012462 | (Hua et al., 2008) |
|  | Coreidae | *Hydaropsis longirostris* | 16,521 | NC_012456 | (Hua et al., 2008) |
|  | Rhopalidae | *Aeschyntelus notatus* | 14,532 | NC_012446* | (Hua et al., 2008) |
|  |  | *Stictopleurus subviridis* | 15,319 | NC_012888 | (Hua et al., 2009) |
|  |  | *Corizus tetraspilus* | 14,989 | KM983397 | (Yuan et al., 2015b) |
|  | Lygaeoidea |  |  |  |  |
|  | Berytidae | *Yemmalysus parallelus* | 15,747 | NC_012464 | (Hua et al., 2008) |
|  | Colobathristidae | *Phaenacantha marcida* | 14,540 | NC_012460* | (Hua et al., 2008) |
|  | Geocoridae | *Geocoris pallidipennis* | 14,592 | NC_012424* | (Hua et al., 2008) |
|  | Lygaeidae | *Kleidocerys resedae resedae* | 14,688 | KJ584365 | (Li et al., 2015b) |
|  | Malcidae | *Chauliops fallax* | 15,739 | NC_020772 | (Li et al., 2013) |
|  |  | *Malcus inconspicuus* | 15,575 | NC_012458 | (Hua et al., 2008) |
|  | Pentatomoidea |  |  |  |  |
|  | Cydnidae | *Macroscytus gibbulus* | 14,620 | EU427338* | (Hua et al., 2008) |
|  | Dinidoridae | *Coridius chinensis* | 14,648 | JQ739179* | (Liu et al., 2012) |
|  | Pentatomidae | *Dolycoris baccarum* | 16,549 | NC_020373 | (Zhang et al., 2013) |
|  |  | *Eurydema gebleri* | 16,005 | NC_027489 | (Yuan et al., 2015a) |
|  |  | *Halyomorpha halys* | 16,518 | NC_013272 | (Lee et al., 2009) |
|  |  | *Nezara viridula* | 16,889 | NC_011755 | (Hua et al., 2008) |
|  |  | *Rubiconia intermedia* | 14,967 | KP207596* | (Yuan et al., 2015a) |
|  | Plataspidae | *Coptosoma bifaria* | 16,179 | NC_012449 | (Hua et al., 2008) |
|  |  | *Megacopta cribraria* | 15,647 | NC_015342 | Direct Submission |
|  | Tessaratomidae | *Eusthenes cupreus* | 16,229 | NC_022449 | (Song et al., 2013) |
|  | Urostylididae | *Urochela quadrinotata* | 16,587 | NC_020144 | (Dai et al., 2012) |
|  | Pyrrhocoroidea |  |  |  |  |
|  | Largidae | *Physopelta gutta* | 14,935 | NC_012432 | (Hua et al., 2008) |
|  | Pyrrhocoridae | *Dysdercus cingulatus* | 16,249 | NC_012421 | (Hua et al., 2008) |
|  |  | *Pyrrhocoris tibialis* | 16,577 |  | This study |

*Incomplete mitochondrial genome.

Dai, Y.T., Li, H., Jiang, P., Song, F., Ye, Z., Yuan, X.Q., Dai, X., Chang, J. and Cai, W.Z., 2012. Sequence and organization of the mitochondrial genome of an urostylidid bug, *Urochela quadrinotata* Reuter (Hemiptera: Urostylididae). Entomotaxonomia 34, 613-623.

Hua, J.M., Dong, P.Z., Li, M., Cui, Y., Zhu, W.B., Xie, Q. and Bu, W.J., 2009. The analysis of mitochondrial genome of *Stictopleurus subviridis* Hsiao (Insecta: Hemiptera-Heteroptera: Rhopalidae). Acta Zootaxonomica Sinica 34, 1-9.

Hua, J.M., Li, M., Dong, P.Z., Cui, Y., Xie, Q. and Bu, W.J., 2008. Comparative and phylogenomic studies on the mitochondrial genomes of Pentatomomorpha (Insecta: Hemiptera: Heteroptera). BMC Genomics 9, 610.

Lee, W., Kang, J., Jung, C., Hoelmer, K. and Lee, S., 2009. Complete mitochondrial genome of brown marmorated stink bug *Halyomorpha halys* (Hemiptera: Pentatomidae), and phylogenetic relationships of hemipteran suborders. Mol. Cells 28, 155-165.

Li, H., Shi, A., Song, F. and Cai, W., 2015a. Complete mitochondrial genome of the flat bug *Brachyrhynchus hsiaoi* (Hemiptera: Aradidae). Mitochondrial DNA, In press.

Li, T., Gao, C., Cui, Y., Xie, Q. and Bu, W., 2013. The complete mitochondrial genome of the stalk-eyed bug *Chauliops fallax* Scott, and the monophyly of Malcidae (Hemiptera: Heteroptera). PLoS ONE 8, e55381.

Li, T., Yi, W., Zhang, H., Xie, Q. and Bu, W., 2015b. Complete mitochondrial genome of the birch catkin bug *Kleidocerys resedae resedae*, as the first representative from the family Lygaeidae (Hemiptera: Heteroptera: Lygaeoidea). Mitochondrial DNA, In press.

Liu, L., Li, H., Song, F., Song, W., Dai, X., Chang, J. and Cai, W.Z., 2012. The mitochondrial genome of *Coridius chinensis* (Hemiptera: Dinidoridae). Zootaxa 3537, 29-40.

Shi, A.M., Li, H., Bai, X.S., Dai, X., Chang, J., Guilbert, E. and Cai, W.Z., 2012. The complete mitochondrial genome of the flat bug *Aradacanthia heissi* (Hemiptera: Aradidae). Zootaxa 3238, 23-38.

Song, W., Li, H., Song, F., Liu, L., Wang, P., Xun, H.Z., Dai, X., Chang, J. and Cai, W.Z., 2013. The complete mitochondrial genome of a tessaratomid bug, *Eusthenes cupreus* (Hemiptera: Heteroptera: Pentatomomorpha: Tessaratomidae). Zootaxa 3620, 260-272.

Wang, P., Li, H., Wang, Y., Zhang, J.H., Dai, X., Chang, J., Hu, B.W. and Cai, W.Z., 2014. The mitochondrial genome of the plant bug *Apolygus lucorum* (Hemiptera: Miridae): presently known as the smallest in Heteroptera. Insect Sci. 21, 159-73.

Yuan, M.L., Zhang, Q.L., Guo, Z.L., Wang, J. and Shen, Y.Y., 2015a. Comparative mitogenomic analysis of the superfamily Pentatomoidea (Insecta: Hemiptera: Heteroptera) and phylogenetic implications. BMC Genomics 16, 460.

Yuan, M.L., Zhang, Q.L., Guo, Z.L., Wang, J. and Shen, Y.Y., 2015b. The complete mitochondrial genome of *Corizus tetraspilus* (Hemiptera: Rhopalidae) and phylogenetic analysis of Pentatomomorpha. PLoS ONE 10, e0129003.

Zhang, Q.L., Yuan, M.L. and Shen, Y.Y., 2013. The complete mitochondrial genome of *Dolycoris baccarum* (Insecta: Hemiptera: Pentatomidae). Mitochondrial DNA 24, 469-71.

Table S3. Saturation test implemented in DAMBE.

| Gene regions | Symmetrical tree | | |  |
| --- | --- | --- | --- | --- |
|  | *Iss* | *Iss.c* | *P* | |
| all positions of 13 PCGs | 0.3837 | 0.8247 | < 0.0001 | |
| 1st positions of 13 PCGs | 0.3392 | 0.8010 | < 0.0001 | |
| 2nd positions of 13 PCGs | 0.2027 | 0.8010 | < 0.0001 | |
| 3rd positions of 13 PCGs | 0.6806 | 0.8010 | < 0.0001 | |

Table S4 The best partitioning schemes and substitution models selected by PartitionFinder for the P123 and AA datasets.

| Data matrix | Subset | Best-fit scheme | Model |
| --- | --- | --- | --- |
| P123 | P1 | *atp6*_pos1, *atp8*_pos1, *nad2*_pos1, *nad3*_pos1, *nad6*_pos1 | GTR+I+G |
|  | P2 | *atp6*_pos2, *atp8*_pos2, *cob*_pos2, *cox1*_pos2, *cox2*_pos2, *cox3*_pos2, *nad1*_pos2, *nad2*_pos2, *nad3*_pos2, *nad4L*_pos2, *nad4*_pos2, *nad5*_pos2, *nad6*_pos2 | GTR+I+G |
|  | P3 | *atp6*_pos3, *atp8*_pos3, *cob*_pos3, *cox1*_pos3, *cox2*_pos3, *cox3*_pos3, *nad2*_pos3, *nad3*_pos3, *nad6*_pos3 | GTR+I+G |
|  | P4 | *cob*_pos1, *cox1*_pos1, *cox2*_pos1, *cox3*_pos1 | GTR+I+G |
|  | P5 | *nad1*_pos1, *nad4L*_pos1, *nad4*_pos1, *nad5*_pos1 | GTR+I+G |
|  | P6 | *nad1*_pos3, *nad4L*_pos3, *nad4*_pos3, *nad5*_pos3 | GTR+G |
| AA | P1 | *atp6*, *atp8*, *cox2*, *nad2*, *nad3*, *nad6* | MtArt+I+G+F |
|  | P2 | *cob*, *cox1*, *cox3* | MtArt+I+G+F |
|  | P3 | *nad1*, *nad4*, nad4L, *nad5* | MtArt+I+G+F |

Table S5. Annotation and organization of the complete mitochondrial genome of *Pyrrhocoris tibialis*

| Gene | Strand | Position | Length (bp) | Intergenic nucleotides | Start codon | Stop codon | Anticodon |
| --- | --- | --- | --- | --- | --- | --- | --- |
| *trnI* | J | 1-64 | 64 | 0 |  |  | GAU |
| *trnQ* | N | 62-130 | 69 | -3 |  |  | UUG |
| *trnM* | J | 140-206 | 67 | 9 |  |  | CAU |
| *nad2* | J | 207-1193 | 987 | 0 | ATA | TAA |  |
| *trnW* | J | 1195-1257 | 63 | 1 |  |  | UCA |
| *trnC* | N | 1250-1312 | 63 | -8 |  |  | GCA |
| *trnY* | N | 1314-1377 | 64 | 1 |  |  | GUA |
| *cox1* | J | 1380-2913 | 1534 | 2 | TTG | T |  |
| *trnL2*(UUR) | J | 2914-2979 | 66 | 0 |  |  | UAA |
| *cox2* | J | 2980-3658 | 679 | 0 | ATG | T |  |
| *trnK* | J | 3659-3730 | 72 | 0 |  |  | CUU |
| *trnD* | J | 3730-3793 | 64 | -1 |  |  | GUC |
| *atp8* | J | 3794-3949 | 156 | 0 | ATA | TAA |  |
| *atp6* | J | 3943-4610 | 668 | -7 | ATG | TA |  |
| *cox3* | J | 4611-5398 | 788 | 0 | ATG | TA |  |
| *trnG* | J | 5399-5462 | 64 | 0 |  |  | UCC |
| *nad3* | J | 5465-5818 | 354 | 2 | ATA | TAA |  |
| *trnA* | J | 5823-5885 | 63 | 4 |  |  | UGC |
| *trnR* | J | 5890-5952 | 63 | 4 |  |  | UCG |
| *trnN* | J | 5957-6024 | 68 | 4 |  |  | GUU |
| *trnS1*(AGN) | J | 6025-6093 | 69 | 0 |  |  | GCU |
| *trnE* | J | 6093-6159 | 67 | -1 |  |  | UUC |
| *trnF* | N | 6161-6227 | 67 | 1 |  |  | GAA |
| *nad5* | N | 6228-7933 | 1706 | 0 | ATA | TA |  |
| *trnH* | N | 7936-8000 | 65 | 2 |  |  | GUG |
| *nad4* | N | 8008-9330 | 1323 | 7 | ATG | TAA |  |
| *nad4L* | N | 9324-9611 | 288 | -7 | ATA | TAA |  |
| *trnP* | N | 9629-9692 | 64 | 17 |  |  | UGG |
| *trnT* | J | 10038-10101 | 64 | 345 |  |  | UGU |
| *nad6* | J | 10102-10583 | 482 | 0 | ATA | TA |  |
| *cob* | J | 10584-11711 | 1128 | 0 | ATG | TAA |  |
| *trnS2*(UCN) | J | 11721-11790 | 70 | 9 |  |  | UGA |
| *nad1* | N | 11791-12740 | 950 | 0 | ATA | TA |  |
| *trnL1*(CUN) | N | 12735-12800 | 66 | -6 |  |  | UAG |
| *rrnL* | N | 12801-14066 | 1266 | 0 |  |  |  |
| *trnV* | N | 14067-14133 | 67 | 0 |  |  | UAC |
| *rrnS* | N | 14134-14957 | 824 | 0 |  |  |  |
| control region | J | 14958-16577 | 1620 | 0 |  |  |  |

Table S6. Start and stop codons and lengths of protein-coding genes (PCGs), and two rRNA genes from three Pyrrhocoroidea species.

| Gene | *Pyrrhocoris tibialis* | | |  | *Dysdercus cingulatus* | | |  | *Physopelta gutta* | | |
| --- | --- | --- | --- | --- | --- | --- | --- | --- | --- | --- | --- |
|  | Start codon | Stop codon | Size (bp) |  | Start codon | Stop codon | Size (bp) |  | Start codon | Stop codon | Size (bp) |
| *atp6* | ATG | TA | 668 |  | ATG | TAA | 666 |  | ATG | TAA | 666 |
| *atp8* | ATA | TAA | 156 |  | ATT | TAA | 156 |  | ATA | TAA | 156 |
| *cob* | ATG | TAA | 1128 |  | ATG | T | 1126 |  | ATG | T | 1132 |
| *cox1* | TTG | T | 1534 |  | TTG | T | 1534 |  | TTG | T | 1534 |
| *cox2* | ATG | T | 679 |  | ATG | T | 679 |  | ATG | T | 670 |
| *cox3* | ATG | TA | 788 |  | ATG | T | 787 |  | ATG | T | 787 |
| *nad1* | ATA | TA | 950 |  | ATA | TAA | 948 |  | ATT | TAA | 921 |
| *nad2* | ATA | TAA | 987 |  | ATA | TA | 983 |  | ATT | T | 979 |
| *nad3* | ATA | TAA | 354 |  | ATT | TAA | 351 |  | ATA | T | 352 |
| *nad4* | ATG | TAA | 1323 |  | ATG | TAA | 1323 |  | ATG | TAA | 1323 |
| *nad4L* | ATA | TAA | 288 |  | ATT | TAA | 282 |  | ATT | TAA | 291 |
| *nad5* | ATA | TA | 1706 |  | ATA | T | 1702 |  | ATT | TAA | 1689 |
| *nad6* | ATA | TA | 482 |  | ATA | TAA | 483 |  | ATT | TAA | 456 |
| *rrnL* | － | － | 1266 |  | － | － | 1268 |  | － | － | 1262 |
| *rrnS* | － | － | 824 |  | － | － | 797 |  | － | － | 779 |

Table S7 Phylogenetic relationships within Lygaeoidea and Pentatomoidea based on different datasets and analytical methods.

| Superfamily | Dataset/method | Phylogeny |
| --- | --- | --- |
| Lygaeoidea | PCG123/MrBayes | ((((Malcidae, Lygaeidae), Geocoridae), Berytidae), Colobathristidae) |
|  | PCG123/RAXML | (((Geocoridae, Berytidae), (Lygaeidae, Malcidae)), Colobathristidae) |
|  | AA/MrBayes | (((Malcidae, Lygaeidae), (Geocoridae, Berytidae)), Colobathristidae) |
|  | AA/RAXML | (((Malcidae, (Lygaeidae, Geocoridae)), Berytidae), Colobathristidae) |
|  | PCG123/Phylobayes | ((Malcidae, Geocoridae, (Lygaeidae, Colobathristidae)), Berytidae) |
|  | AA/Phylobayes | (((Malcidae, Lygaeidae, Colobathristidae), Geocoridae), Berytidae) |
| Pentatomoidea | PCG123/MrBayes | ((Plataspidae, (((Dinidoridae, Tessaratomidae), Cydnidae), Pentatomidae)), Urostylididae) |
|  | PCG123/RAXML | ((Plataspidae, (((Dinidoridae, Tessaratomidae), Cydnidae), Pentatomidae)), Urostylididae) |
|  | AA/MrBayes | (((Plataspidae, ((Dinidoridae, Tessaratomidae), Cydnidae)), Pentatomidae), Urostylididae) |
|  | AA/RAXML | (((Plataspidae, ((Dinidoridae, Tessaratomidae), Cydnidae)), Pentatomidae), Urostylididae) |
|  | PCG123/Phylobayes | ((((Plataspidae, Cydnidae), (Dinidoridae, Tessaratomidae)), Pentatomidae), Urostylididae) |
|  | AA/Phylobayes | (((Plataspidae, ((Dinidoridae, Tessaratomidae), Cydnidae)), Pentatomidae), Urostylididae) |
