# Supplementary figures and images for: The Complete Mitogenome of Pyrrhocoris tibialis (Hemiptera: Pyrrhocoridae) and Phylogenetic Implications
Source: Genes (Basel). 2019 Oct 18;10(10):820. doi: 10.3390/genes10100820 (PMC6826757; doi:10.3390/genes10100820)

***P. tibialis***

1,620 bp

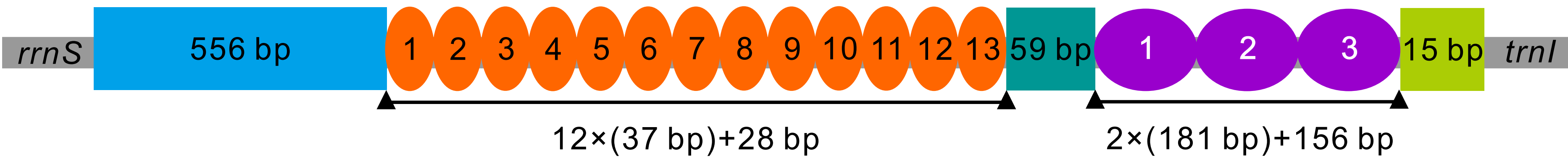

***D. cingulatus***

1,617 bp

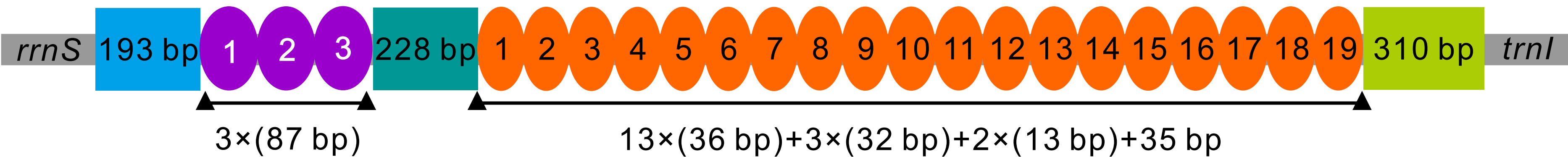

***P. gutta***

224 bp

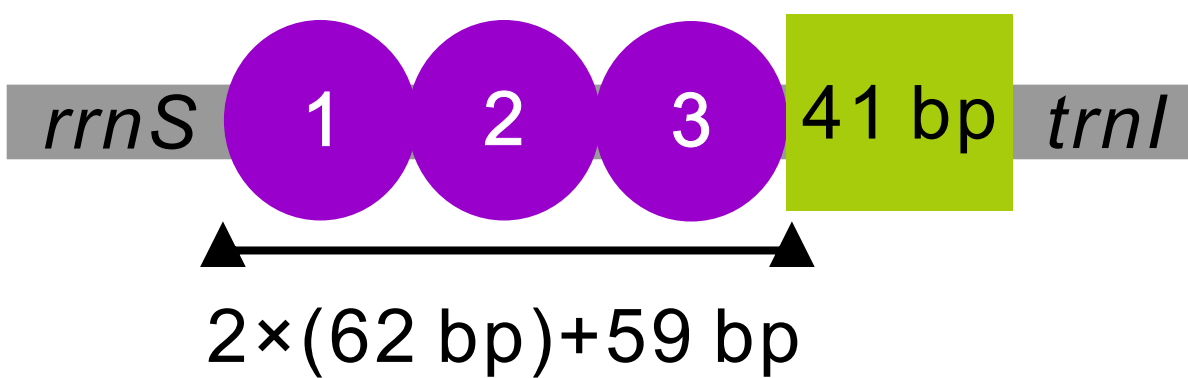

Supplement: Supplementary file 1 [file genes-10-00820-s001.zip › Figure S2.pdf]

A

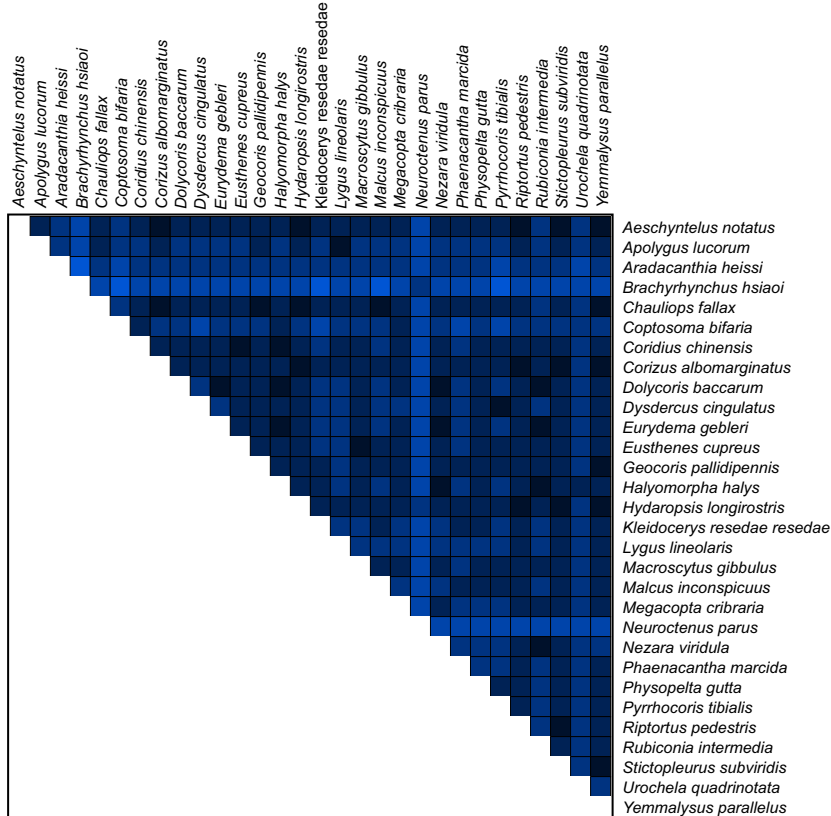

B

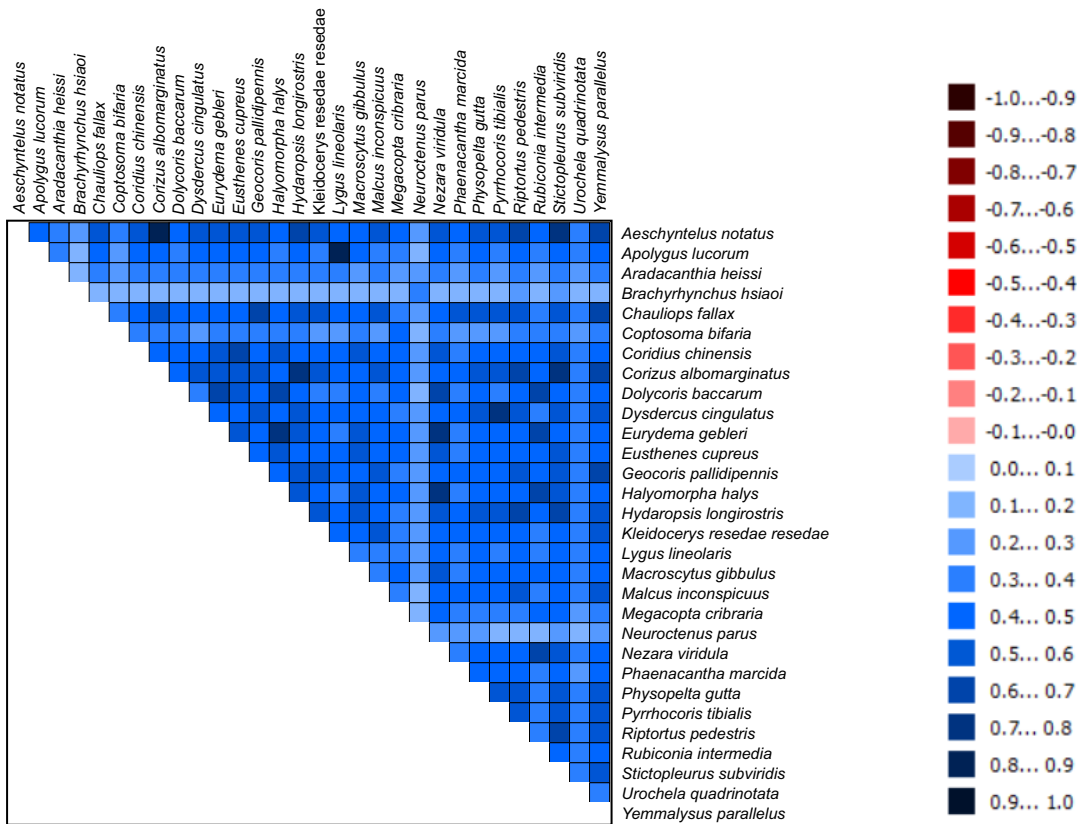

Supplement: Supplementary file 1 [file genes-10-00820-s001.zip › Figure S3.pdf]

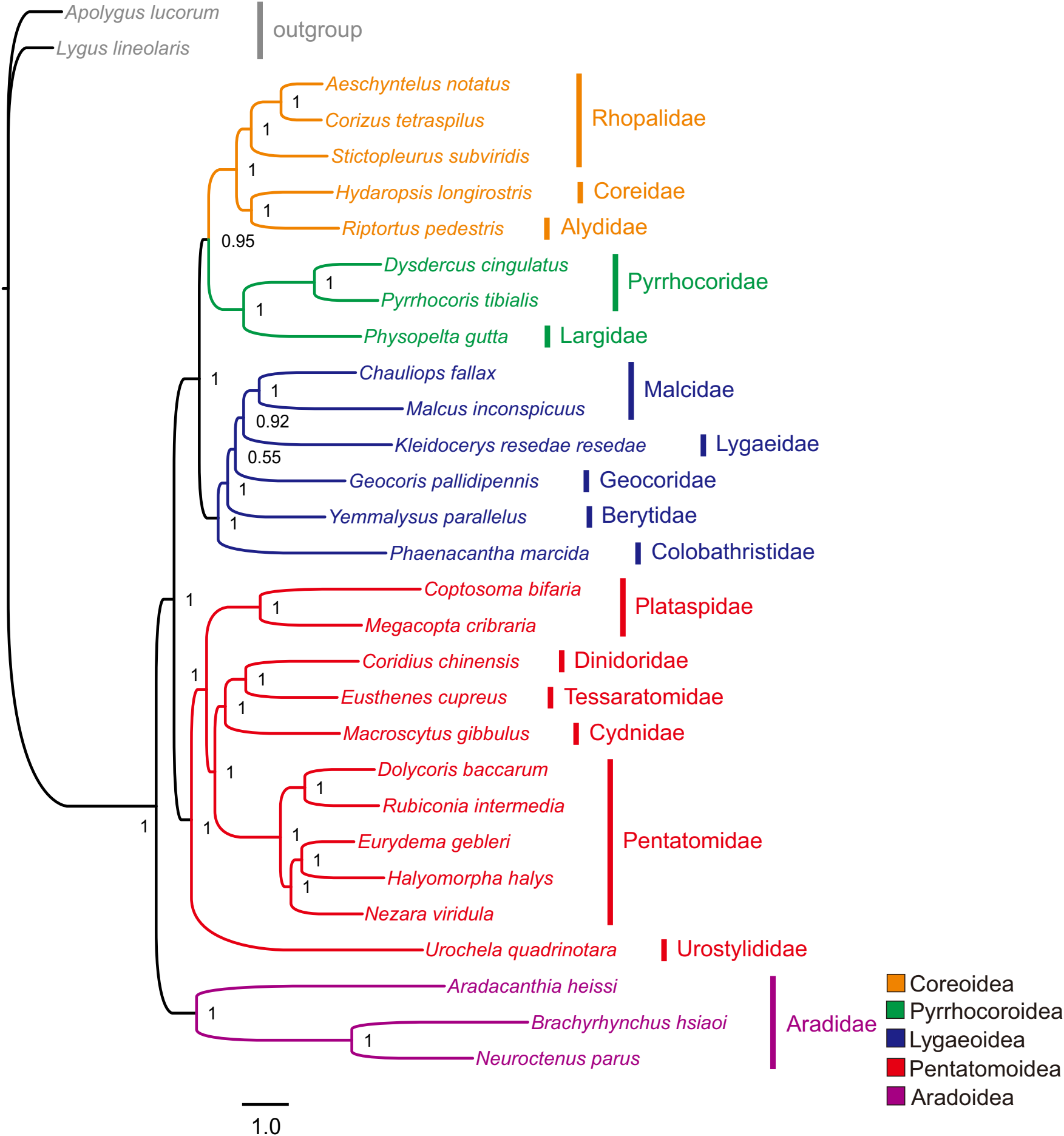

Supplement: Supplementary file 1 [file genes-10-00820-s001.zip › Figure S4.pdf]

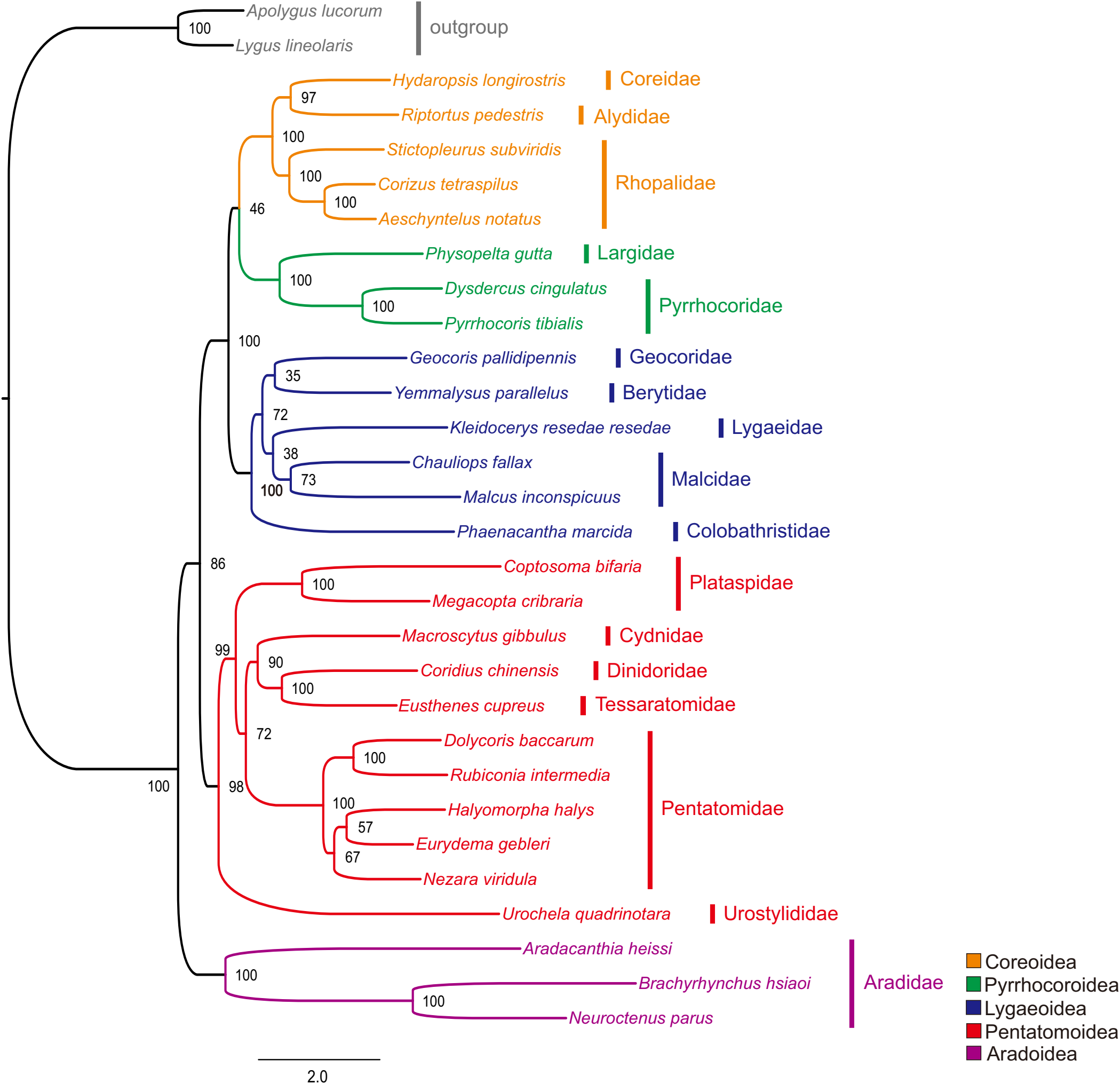

Supplement: Supplementary file 1 [file genes-10-00820-s001.zip › Figure S5.pdf]

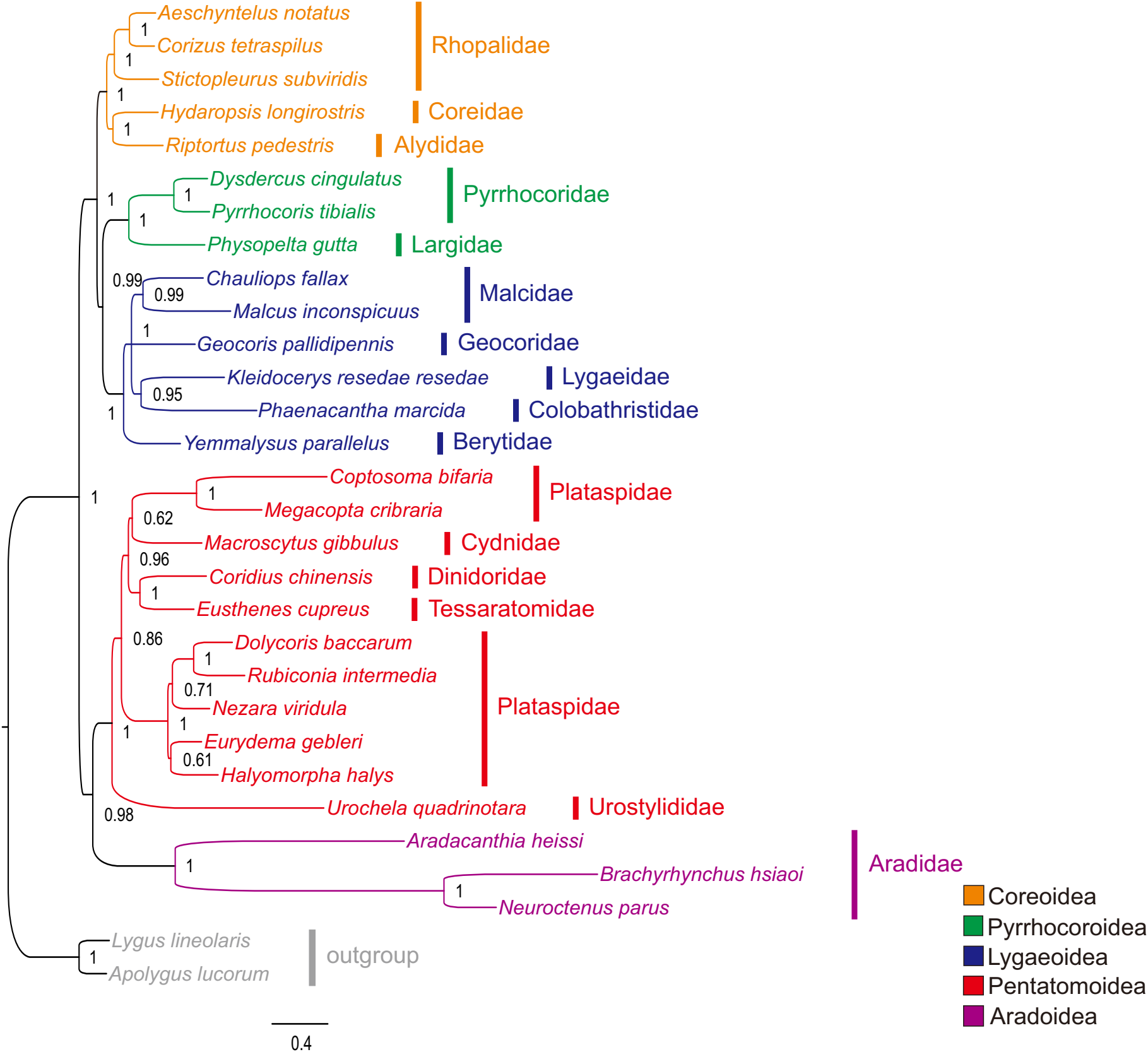

Supplement: Supplementary file 1 [file genes-10-00820-s001.zip › Figure S6.pdf]

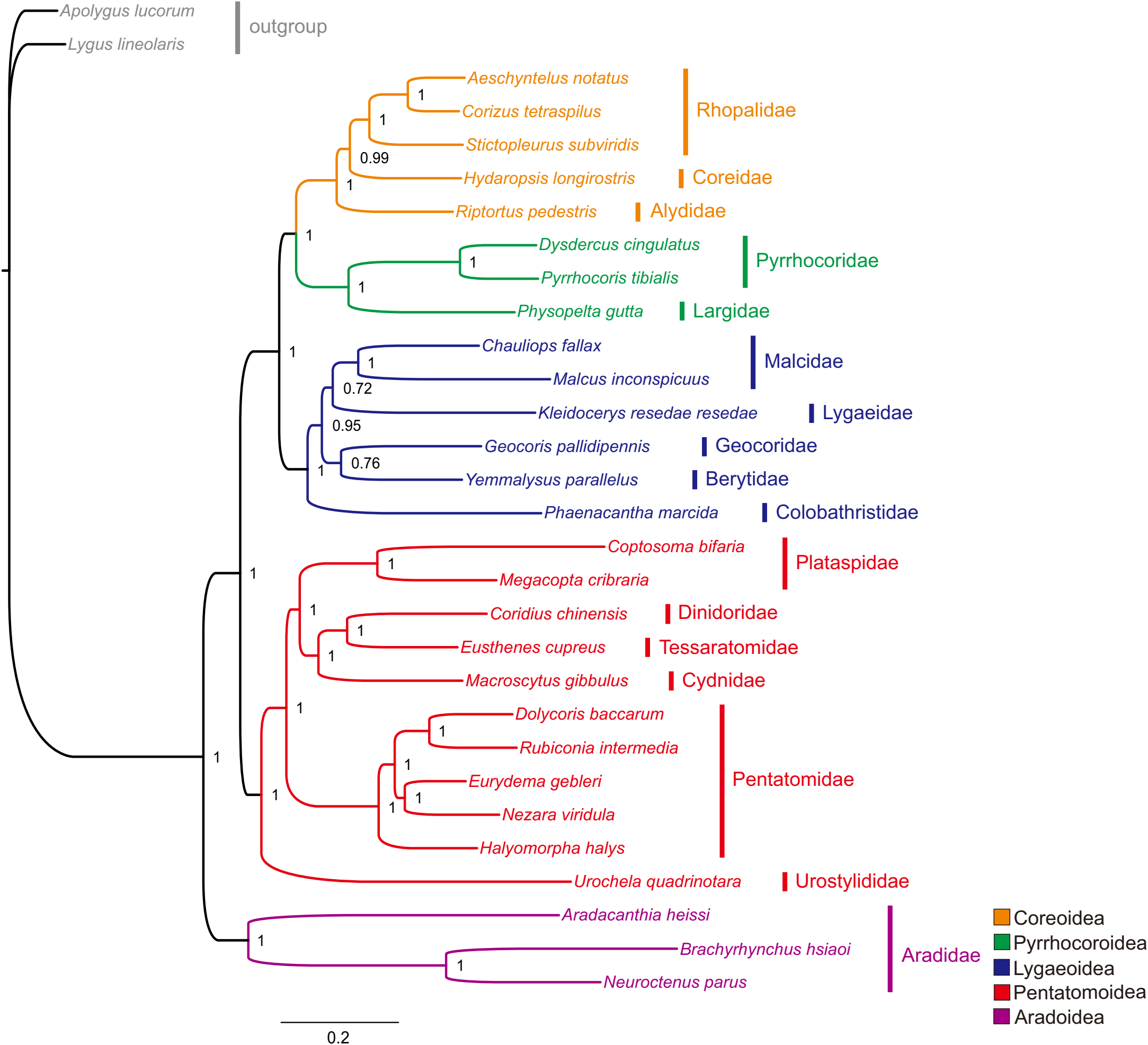

Supplement: Supplementary file 1 [file genes-10-00820-s001.zip › Figure S7.pdf]

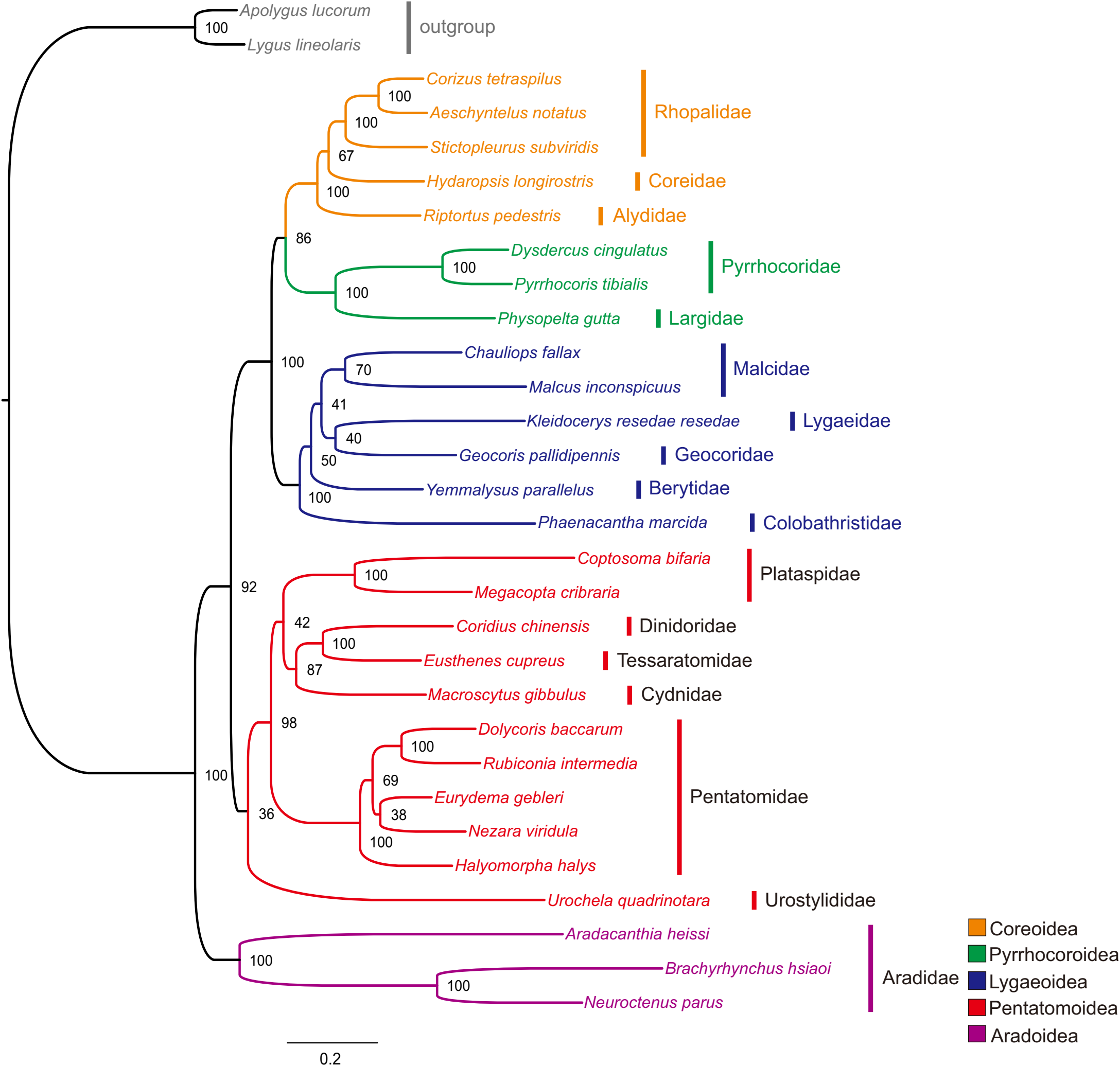

Supplement: Supplementary file 1 [file genes-10-00820-s001.zip › Figure S8.pdf]

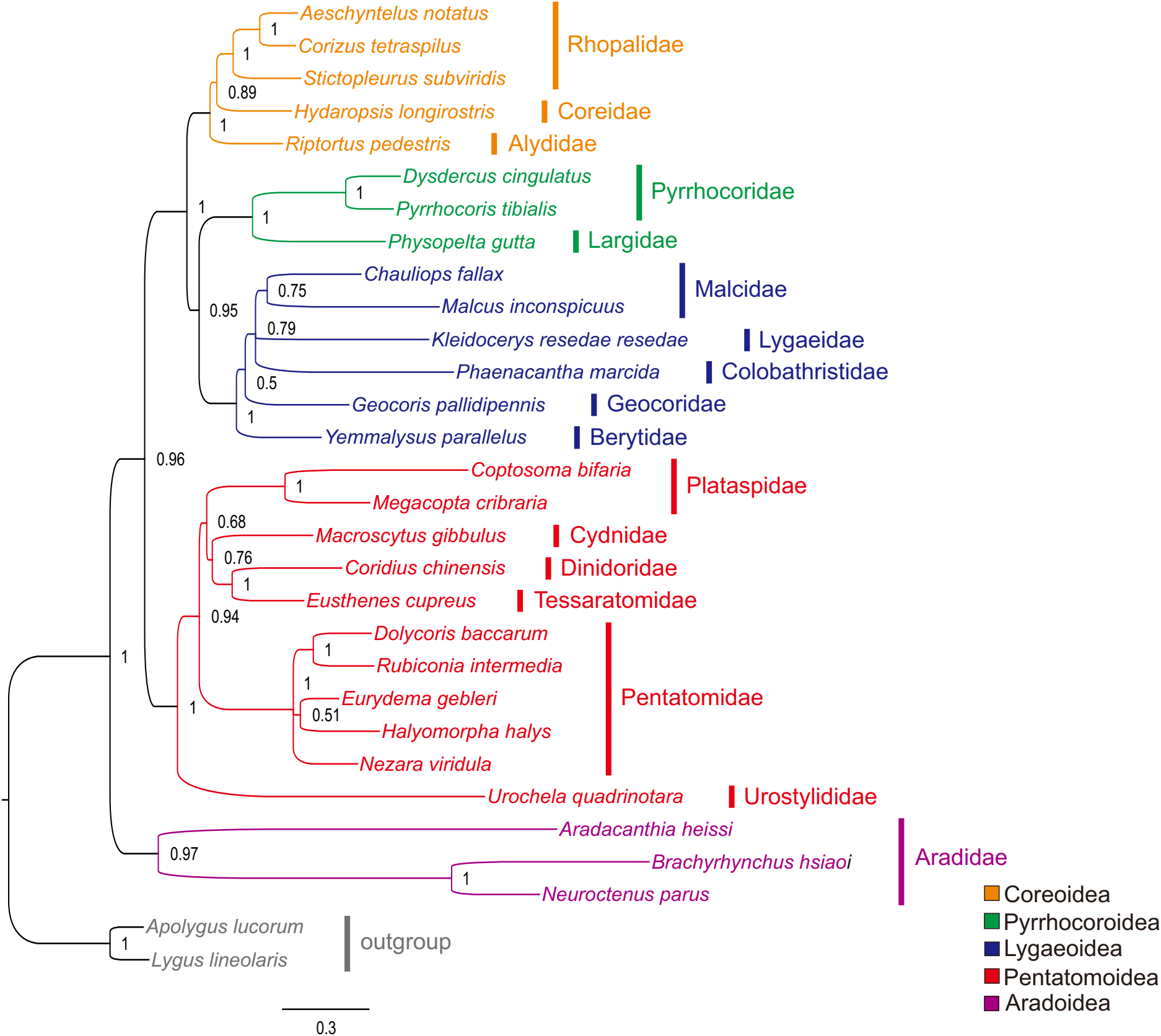

Supplement: Supplementary file 1 [file genes-10-00820-s001.zip › Figure S9.pdf]
